# Saliva in Balancing Oral and Systemic Health, Oral Cancer, and Beyond: A Narrative Review

**DOI:** 10.3390/cancers16244276

**Published:** 2024-12-23

**Authors:** Kohei Okuyama, Souichi Yanamoto

**Affiliations:** 1 Department of Cancer Biology, The University of Texas MD Anderson Cancer Center, 1881 East Rd, Houston, TX 77054, USA; 2 Department of Oral and Maxillofacial Surgical Oncology, Graduate School of Medical and Dental Sciences, Institute of Science Tokyo, Tokyo 113-8549, Japan; 3 Department of Oral Oncology, Graduate School of Biomedical and Health Sciences, Hiroshima University, Hiroshima 734-8553, Japan; syana@hiroshima-u.ac.jp

**Keywords:** oral squamous cell carcinoma, saliva, microRNA, diagnosis, metastasis

## Abstract

Saliva plays a multifaceted role in oral health and systemic well-being. It supports digestion, protects oral tissues, maintains a healthy oral microbiome, and facilitates wound healing. Additionally, saliva serves as a diagnostic tool that reflects systemic health and disease/therapeutic states. Furthermore, although saliva shows a protective effect against oral cancer development, once tumor formation occurs, it may be involved in tumor progression and metastasis via exosomes and microRNAs. This review discusses the essential role of saliva; its relationship with the development, progression, and metastasis of head and neck squamous cell carcinoma (HNSCC); liquid biopsy tools for early diagnosis and monitoring of HNSCC; and the potential of exosomes as therapeutic agents.

## Simple Summary

Saliva is a vital fluid with diverse roles in oral and systemic health, including digestion, oral protection, and microbiome balance and support wound healing. It also serves as a non-invasive diagnostic tool, reflecting overall health and cancer. While saliva can protect against oral cancer, it may promote tumor progression and metastasis through exosomes and microRNA components. This review explores saliva’s biological functions, its role in head and neck cancer progression/metastasis, and the potential therapeutic tool.

## 1. Introduction

Saliva is an essential bodily fluid that performs numerous vital functions. It supports oral health by protecting the teeth through remineralization [1], neutralizing acids [2], and providing antimicrobial agents to prevent infections [3]. Saliva also facilitates digestion by breaking down carbohydrates, enables taste perception by dissolving food molecules, and ensures smooth speech and swallowing owing to its lubricating properties [4]. Additionally, it maintains the integrity of oral tissues and serves as a diagnostic medium that reflects systemic health and disease states [5]. This versatile fluid plays a key role in overall health and well-being.

Head and neck squamous cell carcinoma (HNSCC) is the sixth most common malignancy worldwide and encompasses tumors arising in the oral cavity, nasal cavity, nasopharynx, oropharynx, larynx, hypopharynx, paranasal sinus, and salivary glands, with a global incidence of approximately 880,000 new cases worldwide annually and approximately 450,000 deaths [6]. More than half of the patients with HNSCC are diagnosed at an advanced stage, with an approximate 50% 5-year survival rate [7]. The most common causes of carcinoma include tobacco use, alcohol abuse, nutritional deficiency, poor oral hygiene, and viral infections [8,9]. To date, the molecular mechanisms underlying HNSCC have not been fully explored, and relevant biomarkers to guide therapeutic decisions and evaluate the prognosis of patients with HNSCC are still lacking, which may contribute to the poor outcomes of this disease.

Liquid biopsy using a patient’s saliva is a noninvasive method that may facilitate repeated sampling and real-time monitoring of oral SCC while allowing clinicians to observe the therapeutic response of patients [10]. Physicians and researchers can now obtain effective information from the saliva to overcome tumors, in addition to simple diagnosis. Saliva is an ideal biofluid for liquid biopsies in patients because it is in close proximity to the tumor. In fact, with the increasing incidence of HNSCC, the healthcare system lacks approaches that facilitate early diagnosis. Although the diagnostic and prognostic utility of saliva has been widely recognized, it has yet to be routinely utilized in clinical practice.

The preventive role of saliva in oral SCC development, progression, and metastasis is not yet fully understood. However, it is possible that saliva may have some adverse effects, particularly in oral cancer (OC) progression and metastasis. In this review, the authors summarize the essential and general roles of saliva and the relationship between salivary factors and HNSCC generation, progression, and metastasis. Moreover, this review highlights significant findings on the role of saliva as a biomarker for noninvasive HNSCC diagnosis, the assessment of treatment efficacy, and the development of therapeutic strategies. It emphasizes the clinical implications of integrating multiple biomarkers with clinicopathological findings to enhance diagnostic sensitivity and proposes further studies to explore these correlations.

## 2. Overview of the Essential and General Roles of Saliva

### 2.1. Support for Digestion

**Starch Breakdown:** Amylase, an enzyme known as ptyalin found in saliva, initiates the breakdown of starch into simpler sugars, such as maltose. This preliminary carbohydrate digestion enhances the efficiency of nutrient absorption during the later stages of digestion [11].

**Lubrication of Food and effective chewing:** Saliva moistens food, facilitating easier swallowing and a smoother passage through the digestive tract. This lubrication supports the mechanical aspects of digestion and protects the mucosal surfaces of the digestive system [12].

**Assistance During Hypoglycemia:** It induces increased saliva production. Saliva secretion tends to increase during hypoglycemia, indicating that the body requires energy. Saliva is important in metabolism and energy homeostasis as an early indicator of the body’s need for energy sources. The recent research also implicated that saliva is a useful agent as an alternative sample to the laboratory assessment of hypoglycemic treatment adherence in patients with type II diabetes mellitus [13].

**Support for Taste Perception and Appetite Enhancement:** It can facilitate enhanced taste sensation. Saliva dissolves taste molecules, enabling them to access the taste receptors. This dissolution enhances taste sensitivity and sharpens flavor perception, thereby indirectly boosting appetite and food intake. Saliva deficiency may result in diminished perception of taste [14].

### 2.2. Oral Health Maintenance, Infection Prevention, and Immune Function

**Oxygen and Nutrient Supply:** Saliva contributes to the oral environment by delivering essential oxygen and nutrients to tissues, thereby supporting the maintenance of a healthy oral ecosystem. This nutrient supply aids in the preservation of oral mucosal integrity and overall tissue health [12,15].

**Antimicrobial/Antiviral Properties and Infection Defense:** Saliva contains immunoglobulins, especially immunoglobulin A (IgA), which play a key role in the defense against bacterial [16] and viral entry into the oral cavity. By binding to viral antigens, IgA neutralizes the virus and prevents it from entering host cells. This mechanism is particularly effective in preventing infections caused by respiratory and enteric viruses that enter the body through the oral mucosa [17]. Enzymes and antimicrobial components in the saliva, such as lysozyme, peroxidase [18], and lactoferrin [19], actively inhibit pathogenic bacteria, reduce the risk of oral infections, and contribute to innate immune responses. They also contribute to antiviral defense by disrupting viral structures and inhibiting viral replication [18].

Saliva also contains additional antimicrobial proteins, such as histatins and defensins, which inhibit the growth of oral fungi and bacteria, thereby contributing to infection prevention. These proteins are particularly effective in suppressing the growth of Candida species, thereby reducing the risk of oral candidiasis [20].

**pH Balance Maintenance:** By maintaining a near-neutral pH, saliva prevents acid-induced demineralization of tooth enamel. Saliva acts as a buffer for the oral environment by diluting acidic foods and beverages, thereby reducing potential damage to teeth and mucosal tissues. In addition, the alkaline components of saliva neutralize acids, supporting a balanced pH in the oral cavity [21].

Saliva also protects the oral mucosa from spice-rich food components. Saliva reduces the impact of these potential irritants by coating and lubricating mucosal surfaces, thereby alleviating discomfort from strongly flavored foods [22].

**Anti-inflammatory Effects:** Certain components of saliva, such as melatonin, modulate inflammatory responses and support oral immune function. This anti-inflammatory action helps reduce the risk of oral inflammatory diseases, such as periodontitis and stomatitis, thus contributing to oral and systemic immune health [23]. The interaction of saliva with blood and lymphatic fluids also assists in fluid circulation, further strengthening oral immunity.

**Self-cleaning and Maintenance Cleaning:** Saliva enhances the self-cleaning mechanism of the mouth by washing away food debris and bacteria, thereby maintaining oral hygiene. This “self-cleansing function” helps suppress bacterial growth and supports the health of the oral environment [24,25].

The multiple protective roles of saliva underscore its critical role in maintaining oral health, preventing infections by bacteria and viruses, and modulating immune responses, with broad implications for systemic health.

### 2.3. Regulation of Microbial Competition

**Maintaining Microbial Balance in the Oral Cavity:** Saliva components, such as lactoferrin, also play a significant role in maintaining microbial balance in the oral cavity by inhibiting the growth of specific pathogenic bacteria and fungi. Lactoferrin binds to iron, which is essential for microbial growth, thereby reducing its availability to harmful microorganisms and supporting a healthy oral microbiome [26].

**Nutritional Support for Beneficial Oral Microbes:** Saliva serves as an important source of nutrients for beneficial oral bacteria. It contains amino acids and sugars that support the metabolic activities of these bacteria. Saliva provides a favorable environment for beneficial microbes and contributes to the maintenance of a balanced microbiome, which is crucial for oral health and disease prevention [27].

These findings demonstrate that saliva plays a crucial role in defending against infections and supports the overall health of the oral microbiome by controlling microbial growth and providing essential nutrients to beneficial bacteria.

### 2.4. Protection and Lubrication of the Oral Mucosa

**Mucosal Protection from Dryness and Irritation:** Saliva acts as a protective layer over the mucosa, shielding it from dryness and potential irritants. Mucins, the primary glycoproteins in saliva, provide a viscous layer that coats mucosal surfaces, forming a barrier that protects against mechanical and chemical irritation.

Saliva prevents dryness in the oral cavity and reduces bacterial adherence, thereby lowering the risk of dry mouth-related conditions. The moisturizing effect contributes to a supportive environment for speaking [28].

**Support for Voice Formation:** Saliva keeps the throat lubricated during phonation, facilitating smooth vocal formation. Insufficient saliva can lead to throat dryness, resulting in hoarseness and difficulties in voice projection with increasing phonation threshold pressure [29].

**Prevention of Pathogen Adherence:** Saliva forms a protective barrier, known as oral pellicle, on the oral mucosa that prevents pathogens from adhering to oral surfaces. Mucins and other viscous components in the saliva capture pathogens, reducing their ability to establish themselves on mucosal surfaces. This mechanism aids in preventing infections and supports a healthier oral microbiome balance [30].

The protective and lubricative functions of saliva underscore its essential role in oral comfort during eating and speaking and even vocal quality, and they emphasize its essential role in maintaining oral hygiene and preventing pathogenic colonization. These findings highlight the importance of adequate salivary function in maintaining mucosal integrity and overall quality of life.

### 2.5. Wound Healing and Tissue Protection

**Prevention of Tissue Damage in the Oral Cavity:** Components within saliva exhibit antioxidant properties, effectively reducing oxidative reactions and the production of free radicals within the oral environment [31]. This protective action helps prevent cellular and tissue damage, thereby supporting the integrity of oral structures.

**Promotion of Tissue/Wound Repair Through Growth Factors:** Saliva contains growth factors, such as epidermal growth factor (EGF), which accelerate tissue repair. These factors promote the healing of minor wounds and inflammation in the oral cavity, facilitating rapid recovery from minor injuries or irritation. Salivary EGF contributes to the cellular proliferation and repair process, accelerating wound healing in the oral cavity. This process allows for the recovery of oral wounds at a relatively fast rate, which is particularly beneficial in a dynamic oral environment [32]. Salivary histatins also play a crucial role in wound healing by aiding tissue regeneration and maintaining oral health. These proteins exhibit antimicrobial and cell regenerative properties, making them essential for the maintenance and repair of oral tissues [33].

The healing and protective properties of saliva underscore its critical role in maintaining a rapid oral recovery from minor injuries and inflammation. Saliva enhances the resilience and healing capacity of oral tissues by supporting cellular repair.

### 2.6. Excretion of Hormones and Metabolites

**Presence of Hormones and Metabolites in Saliva:** Saliva contains small amounts of hormones and metabolic byproducts (e.g., steroids), which reflect the body’s internal state. The concentrations of these substances can provide insights into physiological conditions, such as stress levels or metabolic changes [34].

**Noninvasive Diagnostic Tool:** This characteristic of saliva has led to its development as a noninvasive diagnostic tool. Salivary testing has been explored for monitoring stress hormones (such as cortisol) and hormonal balance and offers a potential alternative to invasive diagnostic methods. Studies have demonstrated the feasibility of using saliva to track these biomarkers, making saliva a promising approach for clinical monitoring [35,36].

These findings highlight the potential of saliva as a reliable medium for assessing hormonal and metabolic status, with applications in stress management, disease monitoring, and overall health diagnostics.

### 2.7. Contribution to Psychological Stability

Autonomic Nervous System Influence on Salivation: Salivation is regulated by the autonomic nervous system and is thus closely associated with psychological stress and relaxation states. During stress, salivary secretion tends to decrease, which can lead to a dry mouth; however, ensuring adequate salivation can help alleviate dryness and contribute to stress reduction.

Stress Response Monitoring: Saliva contains cortisol and α-amylase, a key stress hormone and protein, and their levels reflect the body/psychological stress response. Changes in cortisol levels and salivary pH can be monitored using saliva, making saliva a useful biomarker for tracking psychological stress and stress-induced physiological changes [37]. Thus, salivary tests can provide a noninvasive way to monitor stress levels, offering a convenient method for assessing psychological stress without the need for more invasive procedures. Salivation increases during relaxed states, and an abundance of saliva promotes comfort and a sense of security, helping maintain psychological relaxation. This aspect underscores the role of saliva in physiological functions and in supporting mental well-being.

These findings emphasize the integral role of saliva in maintaining psychological stability, providing both a physiological and psychological framework for understanding stress, relaxation, and overall mental health through noninvasive monitoring.

### 2.8. Maintenance of Body Homeostasis

Circadian Rhythm and Salivary Secretion: Salivary secretion follows a diurnal variation corresponding to the body’s circadian rhythm, with different secretion volumes and compositions observed in the morning and evening, including melatonin. This variation in saliva helps the body adapt to different physiological states during the day, providing protective functions that align with the body’s natural rhythms [38].

Blood Pressure Regulation: Specific components of saliva influence blood pressure regulation. Peptides, such as endothelin, which are present in trace amounts in saliva, may contribute to blood pressure modulation through vasoconstriction mechanisms, suggesting a role for saliva in influencing cardiovascular function and maintaining systemic homeostasis [39].

These functions highlight the multifaceted role of saliva in supporting homeostasis through its involvement in the circadian rhythm, pH regulation, and cardiovascular function. The ability of saliva to influence these processes further underscores its significance in maintaining overall physiological stability.

### 2.9. Protection and Strengthening of Teeth

Strengthening Enamel with Calcium, Phosphorus, and Fluoride: Saliva contains important minerals, including calcium, phosphate, and fluoride, which reinforce the enamel and enhance its protection against acids. This strengthens resistance to dental caries and acid erosion. These minerals in saliva help remineralize and repair damaged enamel, restore tooth integrity, and protect teeth from decay [40].

Inhibition of Calculus Formation: Saliva contains specific proteins and enzymes that inhibit the deposition of minerals on teeth, preventing the formation of dental calculus. The presence of salivary proteins, such as proline-rich proteins and statherin, is associated with the prevention of plaque calcification [41].

Maintenance of Sensory Function in Teeth: Saliva plays a crucial role in lubricating the tooth surface, which is essential for maintaining oral sensory function. Dry mouth (xerostomia) can lead to increased tooth sensitivity; however, the lubricating effect of saliva protects teeth from the discomfort caused by sensitivity [42].

Reduction of Dental Hypersensitivity: Saliva coats the tooth surface, helping reduce dental hypersensitivity. When dentin is exposed, saliva forms a protective barrier around sensitive areas, reducing the pain caused by thermal or tactile stimuli [22,43].

**Figure 1:**
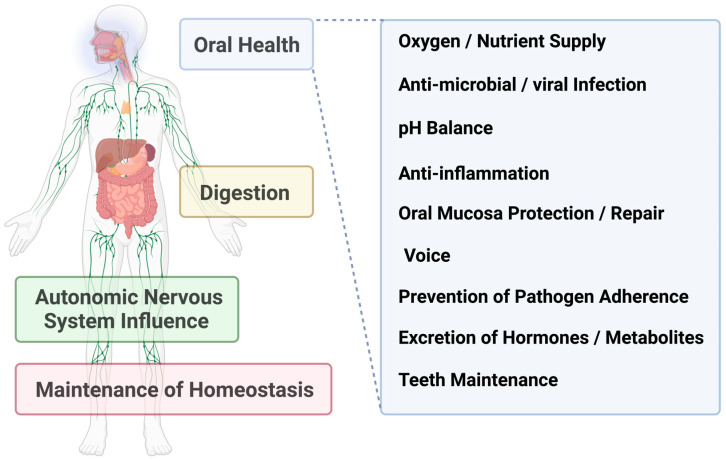
General and essential roles of saliva for oral health and systemic influences.

These findings underscore the protective functions of saliva in maintaining oral health and systemic effects by enhancing enamel strength, preventing calculus formation, and protecting against tooth sensitivity (Figure 1).

### 2.10. Preventive Role of Saliva in Oral Cancer

#### 2.10.1. Oral Cancer Generation Prevention

Saliva’s direct contact with OC lesions makes it a more specific and potentially sensitive screening tool, where >100 salivary biomarkers (DNA, RNA, mRNA, and protein markers) have already been identified, including cytokines (interleukin [IL]-8, IL-1b, and tumor necrosis factor [TNF]-α), defensin-1, p53, Cyfra 21-1, tissue polypeptide-specific antigen, dual specificity phosphatase, spermidine/spermine N1-acetyltransferase, profilin, cofilin-1, and transferrin, which contribute to cancer prevention by removing reactive oxygen species (ROS) generated in the oral cavity [44]. This antioxidant activity helps prevent oxidative damage to cells and tissues, thereby preserving oral health and structural stability and maintaining the health of normal cells [36]. Furthermore, peroxidase, uricase, and superoxide dismutase, which are antioxidant enzymes present in saliva, play a crucial role in neutralizing ROS. These enzymes prevent cellular damage by mitigating oxidative stress, thereby reducing the risk of DNA damage and carcinogenesis [45]. Additionally, the antioxidant components and enzymes in saliva contribute to cancer prevention by breaking down or neutralizing the carcinogenic chemicals present in substances, such as tobacco [46].

Saliva also contains antimicrobial components, such as IgA and lysozyme, which inhibit the proliferation of bacteria and viruses (e.g., Fusobacterium and human papillomavirus) in the oral cavity and are responsible for inducing oral SCC [9]. IgA also prevents inflammation and infection, thereby reducing the risk of chronic inflammation-associated cancer.

Furthermore, saliva is rich in growth factors, such as EGF, which promote wound healing and cellular repair. By facilitating tissue regeneration, these factors help reduce the progression of chronic inflammation in cancer [32]. Saliva plays an essential role in maintaining the health of oral tissues and in suppressing cancer cell development.

#### 2.10.2. Regulation of Oral Microbiome Prevents Oral Cancer Generation

In the regulation of bacterial balance, saliva plays a crucial role in maintaining the microbial balance within the oral cavity. Dysbiosis of the oral microbiome is a risk factor for systemic diseases, including inflammatory disorders and cancers. Notably, *Fusobacterium nucleatum* has been implicated in OC, colorectal cancer, and Alzheimer’s disease [47,48,49]. Moreover, certain products from probiotic bacteria, such as exopolysaccharides derived from *Bacillus subtilis*, are believed to contribute to cancer risk reduction by suppressing inflammation via innate immune system activation. These bacterial products inhibit cancer cell proliferation and promote apoptosis [50,51,52].

Fermentation products of *Lactobacillus paracasei*, such as equol, have been suggested to influence cellular metabolism and inflammatory pathways, potentially reducing cancer risk [53]. These effects include the modulation of oxidative stress, inhibition of pro-inflammatory cytokines, and interactions with signaling pathways, such as nuclear factor-kappa B, which are critical in inflammation-driven carcinogenesis [54]. Additionally, such microbiome-related metabolites may enhance gut microbial diversity and promote the production of beneficial compounds, such as short-chain fatty acids, which further contribute to anticancer effects via activation of antitumor immune system [55]. These findings highlight the potential of microbiome-derived components in cancer prevention.

### 2.11. Role of Saliva in Oral Cancer Progression

#### 2.11.1. Retention of Cancer Risk Factors

Conversely, saliva also acts as a reservoir of carcinogenic substances and facilitates prolonged exposure to these agents. It transiently retains carcinogens from alcohol and tobacco, thereby increasing the risk of prolonged contact with the oral mucosa. Tobacco-derived carcinogens dissolve in saliva and spread across the oral cavity, potentially leading to mucosal damage and an elevated risk of OC [56,57].

#### 2.11.2. Salivary Loss/Decrease-Induced Inflammation Would Affect Cancer Progression

A decrease in salivary secretion or a weakened mucosal barrier in the oral cavity can lead to dryness, increased bacterial proliferation, and prolonged inflammation [58]. Possibly, chronic inflammation of this nature may heighten general risks for cancer development and contribute to cancer cell invasion and progression.

#### 2.11.3. Tissue Repair and Cancer Progression

Growth factors present in saliva, such as EGF, may promote the repair of normal cells but can also enhance the proliferation of cancer cells. In particular, in cases where cancer already exists in the oral cavity, these growth factors may facilitate the growth and invasion of cancer cells, posing an additional risk [59].

#### 2.11.4. Oral Microbiome Disturbance-Derived Modulation for the Oral Environment

Changes in the composition or quantity of saliva can create an environment that favors microorganisms associated with cancer progression. This shift may lead to the dominance of specific bacteria that promote cancer cell growth or metastasis, potentially contributing to the worsening of the disease. As noted, several studies have indicated that *F. nucleatum* is involved in the development of OC. However, whether its expression is specifically increased in OC remains unclear. It has been shown to increase in response to changes in inflammation and immune responses within the oral cavity, acting as a factor that promotes tumor cell proliferation, invasion, and metastasis. Specifically, this bacterium may promote immune evasion in the tumor microenvironment (TME) and support the growth of cancer cells [9,60,61].

Saliva is a multifunctional bodily fluid that may play dual roles in the prevention and progression of OC. Although its antioxidant properties [44], immune support [62], and tissue repair functions [47] contribute to cancer prevention, saliva can also present risks, such as retaining carcinogens that promote chronic inflammation and supporting cancer cell growth through growth factors. Maintaining salivary health and practicing proper oral hygiene are crucial in managing the risk of OC.

### 2.12. Role of Saliva in Oral Cancer Metastasis

#### 2.12.1. Potential Associations Among Saliva, Metastasis Prediction, and TME

Cytokines (e.g., TNF-α, IL-1β, IL-6, and IL-8) and enzymes (lactate dehydrogenase, matrix metalloproteinase (MMP)-9) in saliva are being investigated as biomarkers for the diagnosis and prediction of OC progression [63,64]. Moreover, some combinations of these markers have shown promising sensitivity and specificity in detecting early-stage oral SCC [65]. Inflammatory molecules, immune cells, and the above components in the saliva are thought to influence the preparatory stage of distant metastasis of tumor cells. These molecules are also involved in inflammation and tumor cell migration and have shown promise in predicting the likelihood of metastasis [62,66,67]. However, studies focusing on the specific mechanisms underlying distant metastasis remain limited.

#### 2.12.2. Application of Saliva in Oral Cancer Metastasis Diagnosis

**Figure 2:**
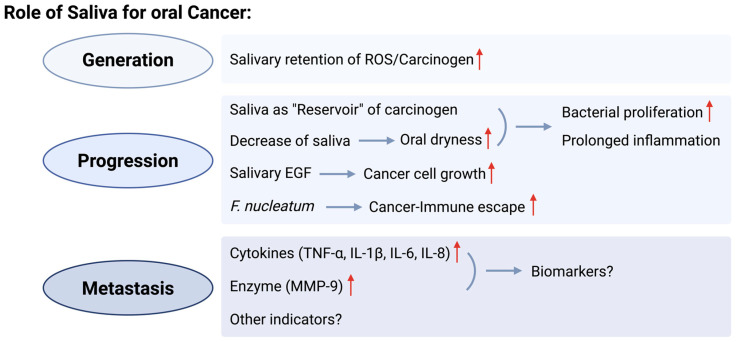
Salivary roles for oral cancer generation, progression, and metastasis. Red arrows indicate rise, increase, or uptake.

In the future, combining the measurement of biomarkers in saliva with current diagnostic imaging tools, such as magnetic resonance imaging or positron emission tomography, has the potential for the early detection and prediction of metastasis. However, specific evidence and directions have not been presented yet, especially using saliva. Further studies are required to elucidate the mechanisms by which saliva contributes to distant metastasis and how this process facilitates tumor progression. Specifically, identifying the role of certain molecules in saliva during various stages of metastasis can lead to the discovery of novel therapeutic targets and the development of preventive strategies (Figure 2).

### 2.13. Saliva as a Liquid Biomarker and Therapeutic Strategies for Oral Cancer Treatment

Saliva has recently garnered significant medical attention as a noninvasive and patient-friendly tool for sample collection. This section summarizes the latest findings on how saliva is utilized for various diagnostic and analytical purposes, the methodologies employed, and how these results are translated into clinical applications to improve patient care. Saliva proteome analysis is a technique that comprehensively analyzes all proteins present in saliva. This includes the identification, quantification, and elucidation of the biological roles of hundreds to thousands of salivary proteins. Through this analysis, physicians and researchers can study how salivary proteins are associated with health and disease states, particularly in OC and other systemic diseases. The primary applications of salivary proteomic analysis include disease diagnosis, monitoring, treatment evaluation, and personalized medicine. Early disease detection is feasible by detecting changes in specific biomarkers in saliva, such as IL-6, MMP-9, and amylase [68]. This approach also enables the tracking of conditions, such as cancer and autoimmune diseases.

Ishikawa et al. investigated salivary metabolite biomarkers by profiling both saliva and tumor tissue samples for OC screening and revealed salivary metabolites showing similar trends in saliva and tissue samples, emphasizing that their approach can help eliminate pseudomolecules that are coincidentally different between OC and healthy states [69]. Moreover, the team further identified salivary metabolomic biomarkers for predicting the prognosis of OC based on comprehensive salivary metabolomics analyses and reported that the salivary concentrations of 5-hydroxylysine and 3-methylhistidine were significant prognostic factors for overall survival in the training group compared with the validation group [70]. Moreover, Winck et al. analyzed the proteome of whole saliva and salivary extracellular vesicles (EVs) from patients with oral SCC and healthy individuals. They identified overrepresented biological processes related to immune responses, peptidase inhibitor activity, iron coordination, and protease binding among differentially expressed proteins. Salivary EVs contain proteins linked to inflammation, metal transport, and cellular growth. The robust proteomics data classified oral SCC with 90% accuracy, highlighting the association of immune processes with oral SCC and the potential of salivary proteomics in determining its prognosis [62].

Although personalized treatment is essential for the future management of OC, the individualization of diagnostic approaches for OC is equally crucial to align with these advancements.

### 2.14. Salivary Cancer-Derived MicroRNA

Saliva also contains genomic information, such as tumor-derived RNA and microRNAs (miRs), which can directly influence tumor cells. miRs contained within tumor-derived exosomes modify gene expression and signaling pathways in normal cells [71]. In addition, they influence fibroblasts and immune cells within the TME, thereby promoting the formation and maintenance of the TME [72,73,74]. Emerging evidence suggests their involvement in promoting tumor growth, invasion, and metastasis. Notably, certain miRNAs suppress these processes. For instance, miR-21 [75,76,77,78,79,80], miR-106b [75], miR-155 (-5p) [76], miR-375 [75,77], and miR-1307-5p [81] function primarily as oncogenic factors that contribute to proliferation, invasion, metastasis, angiogenesis, and the inhibition of apoptosis in tumor cells. MiR-421 from oral SCC cells promotes angiogenesis by targeting heparan sulfate 2-O-sulfotransferase 1 and activating vascular endothelial growth factor (VEGF)/VEGF receptor 2 signaling in endothelial cells, thereby enhancing tumor progression [82]. Moreover, Scholtz et al. revealed that miR-345 and miR-31-5p were consistently upregulated salivary biomarkers for oral SCC, and a three-miRNA panel of miR-345, miR-31-5p, and miR-424-3p could distinguish patients with cancer from control patients with high sensitivity [83]. Conversely, tumor-suppressive miRNAs, such as miR-34a [84], miR-125b [85], miR-133b [86], and miR-422a [76], inhibit tumor progression. However, there is also a small body of evidence supporting the tumor-suppressive effect of miR-21 in breast cancer [87]. Additionally, in normal tissues, these miRs play roles in regulating inflammatory responses and immune activity, thereby influencing the tumor-immune microenvironment [84].

MiRs affect the malignant transformation of normal oral mucosa, fibroblasts, and immune cells within the TME, thereby promoting the establishment of a tumor-supportive niche. Duan et al. reported that the hypoxic HNSCC-derived exosomal miR-5100 promoted the activation of cancer-associated fibroblasts by orchestrating the QKI/AKT/STAT3 axis, which then facilitated HNSCC metastasis and also revealed that miR-5100 derived from plasma exosomes indicated HNSCC malignant progression, suggesting that miR-5100 might be a potential biomarker and therapeutic target for HNSCC [88]. In terms of the immune modulation effect of saliva, Wang et al. summarized the vital role of five immune cells (macrophages, dendritic cells, myeloid-derived suppressor cells, natural killer cells, and T lymphocytes) associated with tumor-derived extracellular vehicles in HNSCC progression and immune escape [89]. Macrophages are considered to be one of the most important immune cells, mainly due to their innate and acquired immune responses to pathogens and prominent positive role in tissue homeostasis, functioning under the miRNA-21-abundance in tumor-derived extracellular vehicles engulfed by CD14 human monocytes, increasing the expression of M2 markers, and inhibiting that of M1 markers, making it easy to drive the progressive tumor milieu. Conversely, the knockout of miR-21 in Snail-expressing HNSCC attenuated snail-induced M2 polarization and inhibited angiogenesis and tumor growth (Table 1) [90].

A recent systematic review and meta-analysis revealed that combined blood- and saliva-derived miR detection demonstrated high diagnostic accuracy for HNSCC [91]. As noted and in line with the proposition by Ishikawa et al., incorporating a secondary sample, such as blood alongside saliva, for miR assessment could potentially lead to a more refined and sensitive diagnostic approach. This finding highlights the need for further detailed prospective clinical trials to validate their efficacy.

### 2.15. Salivary Exosomes as Therapeutic Agents

Individual proteomic profiles derived from saliva facilitate the selection of optimal, patient-specific treatment strategies, making salivary proteomics a cornerstone for advancing diagnostic, treatment monitoring, and therapeutic methodologies [92].

**Figure 3:**
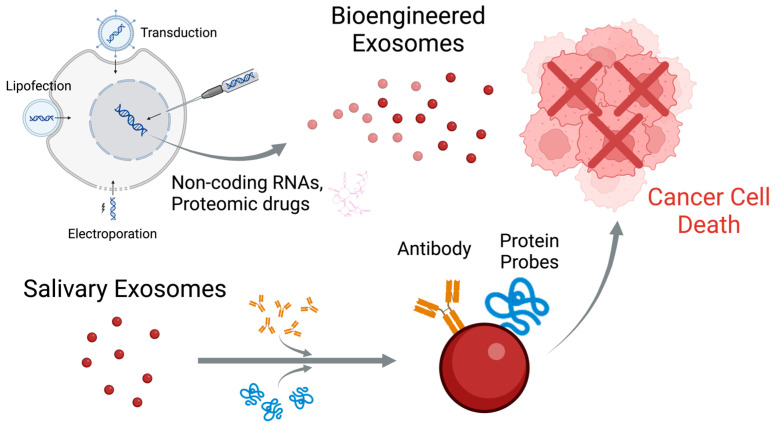
Engineered/Salivary exosomes as potential therapeutic agents.

The recent scientific focus on salivary exosomes as therapeutic agents has been particularly remarkable, and the development of new therapeutic approaches is expected. Recent studies have highlighted the potential of exosomes to deliver therapeutic agents, such as noncoding RNAs or proteins, directly to cancer cells. This ability to transport biological molecules makes exosomes an appealing option for both the diagnosis and targeted treatment of OC. Exosomes derived from OC tissues have shown potential as biomarkers for noninvasive diagnostics because their cargo can reflect tumor characteristics and progression [93]. Additionally, modifications of exosome surface proteins and their cargo have been explored to improve their targeting specificity for OC cells. For example, exosomes from mesenchymal stem cells, engineered to carry tumor-suppressing miRs, have demonstrated the ability to inhibit OC progression. Moreover, the surfaces of exosomes can be modified with antibodies or peptides to enhance their delivery to cancer cells [94]. Exosome-based therapies have also shown promise in overcoming the common challenges of drug delivery systems, such as immune rejection and poor cellular uptake. These exosomes can be engineered to carry therapeutic agents and facilitate their delivery across barriers to reach the tumor site [93] (Figure 3). However, the clinical application of salivary exosomes is still in its infancy, as they have not yet been isolated, purified, or standardized, and reliable diagnostic and therapeutic methods need to be established. Currently, the animal experimental phase has shown the potential to improve exosomes to achieve targeted therapies. However, many challenges remain for clinical trials, including drug transport rate, duration of effect, and in vivo clearance of exosomes [93]. These advancements can lead to more effective and less toxic treatment options than traditional therapies.

## 3. Conclusions and Perspectives

In this review, the authors summarize the normal physiological roles of saliva and explore the microcomponents of saliva that undergo alterations associated with OC. Additionally, the authors discuss the molecular roles of these components in tumor progression and metastasis and provide perspectives on future research directions.

In addition to the abovementioned evidence, valuable saliva-based genetic testing kits allow for the convenient analysis of genetic disease risks, drug metabolism capabilities, and even ancestral information. As a bodily fluid garnering attention beyond the realm of medicine, saliva has the potential to become a powerful tool for personal identification, akin to fingerprints and DNA, and for predicting various diseases and their prognoses. Therefore, the authors suggest that the establishment of a salivary bank might be the same as that of an organ tissue bank because saliva is available as a noninvasive and easily accessible fluid and remains an enigmatic subject in wide biological research. Despite its clinical potential and prospective therapeutic applications, many of its aspects remain unexplored, leaving room for further investigation and innovation [95]. Currently, tumor tissue banks are maintained at various facilities worldwide to support the development of therapeutic strategies and address treatment resistance in different cancer types. Similarly, studies on blood samples are advancing, with an increasing number of institutions establishing blood sample banks. Saliva collected from patients with cancer has the potential to serve as “another liquid sample”, offering a unique perspective to enhance diagnostic accuracy and inform therapeutic decision-making. The establishment of saliva banks can provide invaluable resources for future studies and innovations in cancer treatment.

In conjunction with the establishment of a framework for the systematic collection of these samples, further in-depth research is needed to elucidate the effects of miRs and exosomes on cancer growth, metastasis, and their potential clinical applications. Furthermore, the active implementation of clinical trials aimed at cancer control utilizing these samples is highly desirable.

The biological questions and challenges remain: How do these biomarker molecules enter the body, circulate, and function in the saliva? How do these compounds transition into the bloodstream? These issues unresolved pose significant scientific challenges and should be addressed in future research.

## Figures and Tables

**Table 1:** Salivary miRs and their potential roles for OC generation, generation, and metastasis.

miRNA	Role	Significance	Reference
miR-21	Oncogenic; promotes proliferation, invasion, metastasis, and angiogenesis; inhibits apoptosis	Highly abundant in tumor microenvironment, influences macrophage polarization	[75,76,77,78,79,80]
miR-106b	Oncogenic; contributes to tumor growth and metastasis	Supports malignancy	[75]
miR-155 (-5p)	Oncogenic; facilitates immune modulation and tumor progression	Associated with immune escape	[76]
miR-375	Oncogenic; associated with tumor maintenance	Supports metastasis	[75,77]
miR-1307-5p	Oncogenic; supports tumor proliferation and invasion	Potential therapeutic target	[81]
miR-421	Promotes angiogenesis via VEGF signaling in endothelial cells	Enhances tumor progression	[82]
miR-345	Biomarker; upregulated in oral SCC	Potential diagnostic biomarker	[83]
miR-31-5p	Biomarker; upregulated in oral SCC	Potential diagnostic biomarker	[83]
miR-424-3p	Biomarker; part of diagnostic miRNA panel for oral SCC	Diagnostic sensitivity in combination panel	[83]
miR-34a	Tumor-suppressive; inhibits tumor progression	Regulates inflammatory and immune responses	[84]
miR-125b	Tumor-suppressive; inhibits tumor progression	Regulates immune activity	[85]
miR-133b	Tumor-suppressive; inhibits tumor progression	Contributes to immune modulation	[86]
miR-422a	Tumor-suppressive; inhibits tumor progression	Contributes to immune modulation	[76]
miR-5100	Promotes activation of cancer-associated fibroblasts via QKI/AKT/STAT3 axis	Indicates malignant progression of HNSCC; potential biomarker	[88]
